# *Magnaporthe oryzae* pathotype *Triticum* (MoT) can act as a heterologous expression system for fungal effectors with high transcript abundance in wheat

**DOI:** 10.1038/s41598-022-27030-z

**Published:** 2023-01-03

**Authors:** Cassandra Jensen, Diane G. O. Saunders

**Affiliations:** John Innes Centre, Norwich, NR4 7UH UK

**Keywords:** Next-generation sequencing, Microbiology, Molecular biology, Plant sciences

## Abstract

Plant pathogens deliver effector proteins to reprogramme a host plants circuitry, supporting their own growth and development, whilst thwarting defence responses. A subset of these effectors are termed avirulence factors (Avr) and can be recognised by corresponding host resistance (R) proteins, creating a strong evolutionary pressure on pathogen Avr effectors that favours their modification/deletion to evade the immune response. Hence, identifying Avr effectors and tracking their allele frequencies in a population is critical for understanding the loss of host recognition. However, the current systems available to confirm Avr effector function, particularly for obligate biotrophic fungi, remain limited and challenging. Here, we explored the utility of the genetically tractable wheat blast pathogen *Magnaporthe oryzae* pathotype *Triticum* (MoT) as a suitable heterologous expression system in wheat. Using the recently confirmed wheat stem rust pathogen (*Puccina graminis* f. sp. *tritici*) avirulence effector AvrSr50 as a proof-of-concept, we found that delivery of AvrSr50 via MoT could elicit a visible *Sr50*-dependant cell death phenotype. However, activation of *Sr50*-mediated cell death correlated with a high transgene copy number and transcript abundance in MoT transformants. This illustrates that MoT can act as an effective heterologous delivery system for fungal effectors from distantly related fungal species, but only when enough transgene copies and/or transcript abundance is achieved.

## Introduction

Plant pathogens deliver effector proteins to their hosts to reprogram plant defense circuitry and facilitate parasitic colonization^1^. However, in certain plant genotypes a subset of effector proteins termed avirulence factors (Avr) can be recognised by corresponding host resistance (R) proteins. This interaction triggers a hypersensitive immune response and renders the pathogen avirulent, halting its proliferation^2^. However, host recognition exerts a strong evolutionary pressure on pathogen Avr effectors that favours their modification/deletion to circumvent the immune response^3^. In agriculture, this frequently leads to the emergence of virulent pathogen races, compromising *R*-gene mediated resistance. Identifying Avr effectors and subsequently tracking their allele frequencies in a population is critical for understanding the loss of host recognition. It can also lead to better informed resistance strategies and thereby prolong the longevity of deployed resistance sources^4^. This is particularly important in the case of domesticated crops such as wheat, where resistance breeding is frequently outpaced by pathogen evolution due to the limited genetic base of resistance sources^5^.

To permit widescale monitoring of loss of host recognition, virulence loci must firstly be defined. The genomics era has markedly accelerated the identification of potential virulence loci, with hundreds of candidate effectors being proposed for many agriculturally important fungal pathogens^6,7^. However, subsequent functional confirmation of Vir/Avr activity in the native plant host has remained a largely arduous task, particularly in wheat^8^. As a consequence, the pace of *R*-gene discovery has now considerably outstripped the confirmation of corresponding virulence loci^9^. This is particularly evident for obligate biotrophic fungal pathogens, that are typically incalcitrant to transformation and/or where suitable surrogate systems are lacking^7,8^, constraining Vir/Avr validation. However, recent advances in defining the first three Avr effectors (AvrSr50, AvrSr35 and AvrSr27) for the devastating wheat stem rust pathogen (*Puccinia graminis* f. sp. *tritici*; *Pgt*) is encouraging^10–12^. Although the systems available to subsequently confirm Avr effector function in the wheat host still remain limited and challenging.

In wheat, one strategy to confirm Avr function is to utilise recombinant *barley stripe mosaic virus* (*BSMV*) strains harbouring the candidate Avr effectors of interest. This approach was successfully used to confirm AvrSr50 and AvrSr27 function in wheat, however, it is dependent on the particular varieties being susceptible to the virus and *BSMV* also elicits disease symptoms itself which can eclipse cell death caused by recognition of an Avr effector^13,14^. Another alternative surrogate delivery system for wheat involves utilisation of the bacterial type III secretion system (T3SS) of *Pseudomonas fluorescens*^15^. However, this method has proven inconsistent in its ability to elicit avirulence phenotypes in wheat^14^. Alternative strategies include wheat protoplast assays^14^ and *Agrobacterium*-mediated transient co-expression of the candidate *Avr* and *R* genes in the surrogate plant system *Nicotiana benthamiana*^16^, which are both dependent on the corresponding *R*-gene being cloned.

Recently, *Ustilago hordei*—the causal agent of smut disease on barley and oat—has shown great promise as a heterologous fungal expression system for testing virulence factors in barley^17^. However, similar surrogate fungal systems for use in screening candidate Vir/Avr function in wheat are lacking. Here we set out to determine if the wheat blast pathogen *Magnaporthe oryzae* pathotype *Triticum* (MoT) could act as a suitable heterologous expression system, using the recently confirmed *Pgt* AvrSr50 avirulence protein as a proof-of-concept. MoT is particularly attractive for this purpose, given that the related rice blast pathogen, *M. oryzae*, is a long-standing model system for studying plant–microbe interactions and the breakdown of host resistance^18^. Furthermore, the ease of genetic transformation for *M. oryzae* pathotypes and ability to axenically culture the pathogen, makes MoT particularly amenable as a surrogate effector delivery system in wheat. We found that delivery of *AvrSr50* via MoT could induce an *Sr50-*dependent HR, but only when a high copy number and transcript abundance was achieved. This tends to indicate that MoT could be useful as an effective surrogate delivery system but only when sufficient expression of the transgene is obtained.

## Results

### The *PWL2* promoter effectively drives expression of *AvrRmg8* in MoT

**Figure 1:**
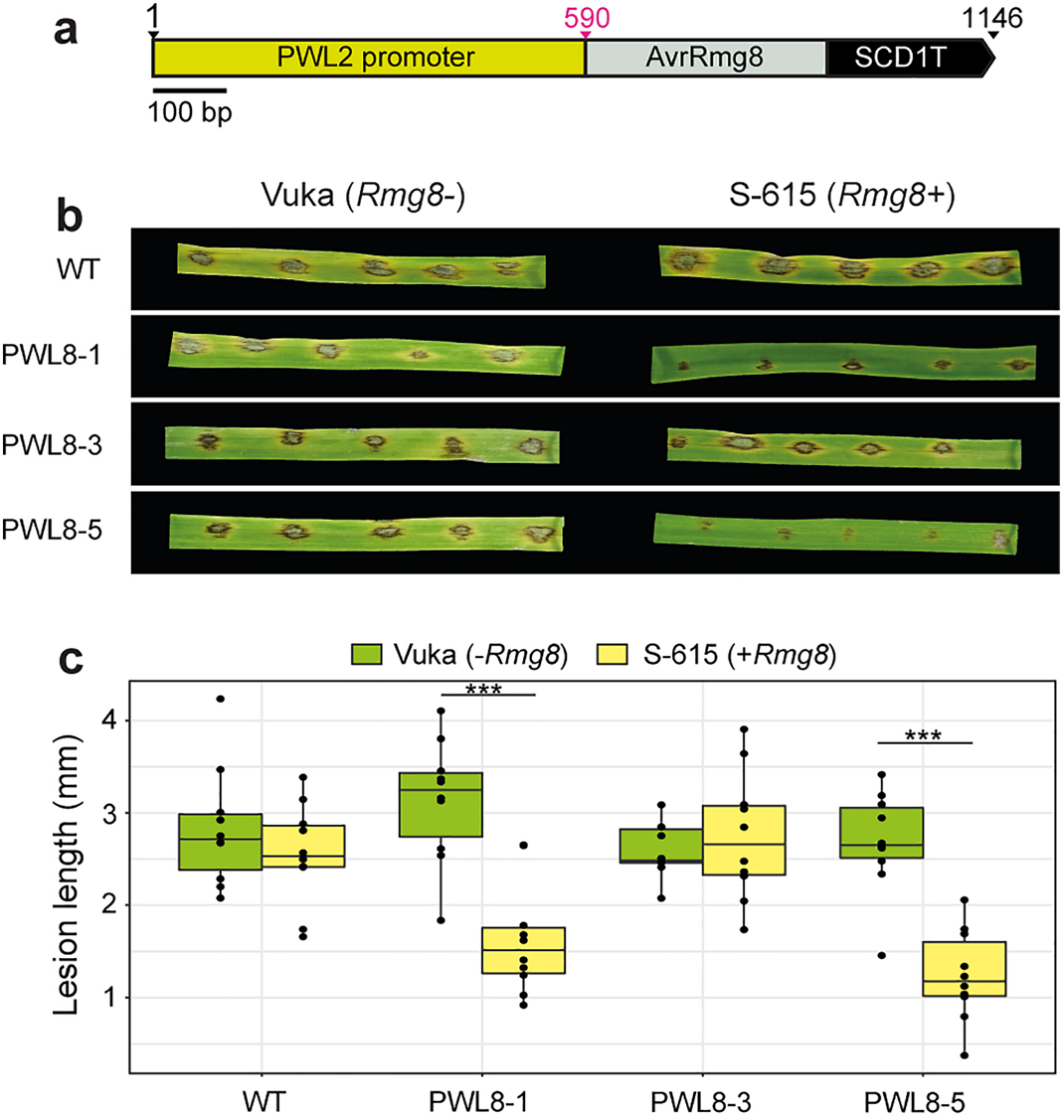
Introduction of *AvrRmg8* into MoT strain N06047 under the *PWL2* promoter induced a visible *Rmg8*-mediated HR phenotype. (**a**) Schematic representation of the pCB-Ppwl2*-*AvrRmg8*-*stop vector used for transformation of MoT (strain N06047). Scale bar represents 100 bp. (**b**,**c**) Introduction of the *PWL2p::AvrRmg8* transgene into MoT induced a visible *Rmg8*-mediated hypersensitive response (HR), specifically restricting the growth of two of the three transformants obtained when inoculated onto the wheat line S-615 (*Rmg8* +). The second leaf of two-week old wheat seedings of the lines Vuka (*Rmg8-*) and S-615 (*Rmg8* +) were inoculated with conidial suspensions derived from three separate transformants (PWL8-1,3,5) using the spot inoculation method. Images were taken and lesion lengths measured at 4 days post-inoculation, with three biological replicates conducted (separate leaves). WT, wild-type (MoT strain N06047). Asterisks denote statistically significant differences (***: *p* < 0.001; 2-tailed *t*-test).

To identify a suitable promoter for driving transgene expression of effectors in MoT, we first considered the promoter from the *PWL2* gene, which is a well-characterised *M. oryzae* effector that is known to be highly expressed during infection^19^. We cloned the 330 bp MoT avirulence effector *AvrRmg8*^20^ (genbank accession LC223814) downstream of the 590 bp *PWL2* promoter in the pCB-Ppwl2-mcherry-stop vector^21^ (Fig. 1a). The resulting pCB-Ppwl2*-*AvrRmg8*-*stop vector was then used for protoplast transformation of the MoT strain N06047. This MoT strain was selected as it is known to be virulent on the wheat line S-615 (*Rmg8* +) and we found carries a previously uncharacterised virulence allele of *AvrRmg8*^22^ (Supplementary Figure S1). A total of six transformants were confirmed by PCR and three positive transformants selected for further analysis. To establish if the *AvrRmg8* gene was expressed sufficiently in the transformants to induce *Rmg8*-mediated HR, the three selected transformants (PWL8-1, PWL8-3 and PWL8-5) were subjected to infection assays on the second leaf of two to three-week old seedlings of the wheat lines Vuka (*Rmg8*-) and S-615 (*Rmg8* +), alongside the wild type MoT N06047 strain. Two of the three transformants (PWL8-1, PWL8-5) displayed a clear visible HR phenotype 4 days post-inoculation (dpi) on *Rmg8* + wheat that was not evident with the wild-type MoT N06047 strain (Fig. 1b). Furthermore, lesions induced by transformants PWL8-1 and PWL8-5 were significantly shorter in length on wheat leaves from the line containing *Rmg8*, when compared to the wild type strain (Fig. 1c). This analysis illustrates that the expression levels achieved using the *PWL2* promoter were sufficient for *AvrRmg8* to induce a visible *Rmg8*-dependent HR phenotype when introduced into the MoT N06047 strain.

### A visible *Sr50*-dependant HR phenotype was not evident in MoT transformants expressing *AvrSr50* under the *PWL2* promoter

To determine if MoT could be used as a surrogate system to heterologously express effector proteins in wheat from distantly related fungal species, we selected the previously confirmed avirulence effector from *Pgt*, *AvrSr50*^11^. As *Magnaporthe* cytoplasmic effector proteins also require a signal peptide (SP) for targeting proteins to the host cytoplasm^23,24^, we introduced the coding sequence for the signal peptide from the MoT *PWT3* effector^25^ downstream of the *PWL2* promoter in the pCB-Ppwl2-mcherry-stop vector^21^. The 54 bp *PWT3 SP* was amplified from MoT strain N06047 and cloned in frame with the 333 bp C-terminal effector domain of *AvrSr50,* without its native signal peptide (Supplementary Figure S2a). Following transformation of MoT strain N06047 with pCB-Ppwl2*-*PWT3SP-AvrSr50*-*stop, a total of three transformants were confirmed by PCR and all transformants selected for subsequent analysis. The three transformants (PWLS-2, PWLS-8 and PWLS-11) were used for infection of the second leaf from two to three-week old seedlings of two wheat lines differential for the corresponding *R* gene, *Sr50*. At 4 to 5 dpi, there was no visible evidence of an *Sr50-*dependent HR for the three tested *PWL2p::PWT3SP::AvrSr50* transformants (Supplementary Figures. S2b and S3). Furthermore, the length of the lesions induced by the three transformants were comparable to the wild-type and between the two wheat lines differential for *Sr50* (Supplementary Figure. S2c). This indicates that the avirulence properties of AvrSr50 could not be detected for the three transformants analysed when *PWL2p::PWT3SP::AvrSr50* was integrated into MoT.

### Integration of *AvrSr50* under the *PWT3* promoter elicited a visible *Sr50*-dependant HR phenotype

**Figure 2:**
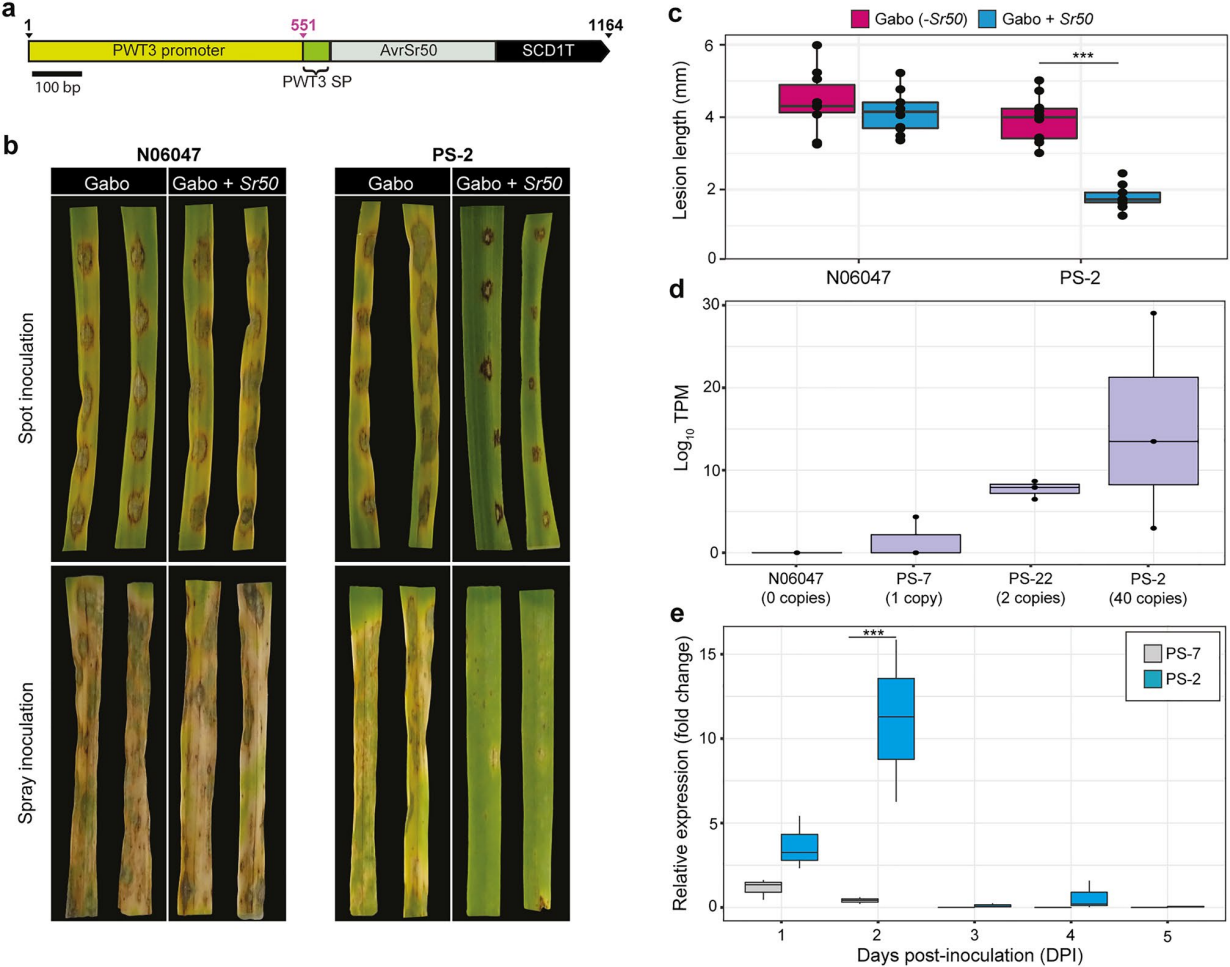
Introduction of *Pgt AvrSr50* into MoT under the *PWT3* promoter and signal peptide induced a visible *Sr50*-mediated HR phenotype when the transgene was highly expressed. (**a**) Schematic representation of the pCB-PWT3p*-*PWT3SP-AvrSr50*-*stop vector used for transformation of MoT (strain N06047). Scale bar represents 100 bp; SP, signal peptide. (**b,c**) Introduction of *PWT3p::PWT3SP::AvrSr50* into MoT induced a visible *Sr50*-mediated hypersensitive response (HR) for one transformant (termed PS-2), specifically restricting its growth when inoculated onto the wheat line Gabo + *Sr50*. Conidial suspensions from the MoT strain N06047 and the PS-2 transformant were inoculated onto the second leaf of two-week old wheat plants (lines Gabo [*Sr50-*] and Gabo + *Sr50*). Inoculations were performed using both the spot and spray inoculation methods and three biological replicates (separate leaves) assessed. Images were taken and lesion lengths analysed at 4 days post-inoculation (dpi). (**d**) *AvrSr50* expression correlated with the number of *AvrSr50* insertion events in MoT. AvrSr50 expression level was determined from RNA-seq analysis of wheat leaves (Gabo *–Sr50*) harvested 3 dpi with three independent transformants PS-7, PS-22, PS-2, and the wild type MoT N06047 strain. Three biological replicates (separate plants) were analysed. Expression data is represented as Log_10_ TPM (transcripts per million). Each data point represents a single biological replicate (separate plants and experiments). (**e**) *AvrSr50* expression peaked at 2 dpi for the PS-2 transformant. Relative expression was assessed by RT-qPCR of wheat leaves (Gabo *-Sr50*) following infection with PS-7 (single copy) and PS-2 (40 copies) transformants. Three biological replicates (separate leaves) were analysed for each time-point. Relative expression is shown as the fold change relative to PS-7 infected leaves at 1 dpi. Asterisks denote statistically significant differences (***: *p* < 0.001; 2-tailed *t*-test).

To explore the possibility that a different promoter could enhance the levels of *AvrSr50* expression sufficiently to elicit an *Sr50-*dependent HR, we chose three further promoters for analysis. This included the MoT 550 bp *PWT3* effector promoter^25^, the 478 bp promoter from the *M. oryzae* ribosomal protein 27 (*RP27*)^23^ and the 357 bp *TrpC* promoter from *Aspergillus nidulans*^26^. We introduced the three promoters upstream of the *PWT3* SP and C-terminal effector domain of *AvrSr50* (Fig. 2a and Supplementary Figure. S4a,b) and each resulting vector was then used for transformation of the MoT N06047 strain. A total of two to nine transformants were confirmed using PCR for each of the three constructs and two transformants selected for the two constitutive promoters (*RP27p*::*PWT3SP::AvrSr50* and *TrpCp::PWT3SP::AvrSr50*) and six transformants for the MoT promoter (*PWT3p::PWT3SP::AvrSr50*). Transformants were then used for infection of leaves from two-week old seedlings of the two wheat lines differential for *Sr50*. For the constitutive promoters (from *RP27* and *TrpC*) none of the transformants induced a visible *Sr50*-dependant HR phenotype (Supplementary Figures. S4c,d,e,f and S3). However, when utilizing the *PWT3* promoter a single transformant (PS-2) was identified that induced a cell death phenotype specifically on the *Sr50* + wheat line (Fig. 2b,c and Supplementary Figure. S5). Accordingly, following spot inoculation lesion lengths were significantly shorter for PS-2 in wheat leaves containing *Sr50*, when compared to the wild-type MoT N06047 strain and to inoculation with PS-2 on the *-Sr50* wheat line (Fig. 2c). In spray inoculations, again the level of infection was substantially reduced for PS-2 in an *Sr50* dependant manner, when compared to the wild-type MoT N06047 strain (Fig. 2b and Supplementary Figure. S3). This illustrates that *PWT3p::PWT3SP::AvrSr50*-expressing MoT transformants can induce an *Sr50*-dependant HR phenotype.

### Expression levels of the *PWT3p::PWT3SP::AvrSr50* transgene correlated with induction of the *Sr50*-dependant HR phenotype

To further explore why only a single *PWT3p::PWT3SP::AvrSr50* MoT transformant was found that elicited an *Sr50*-dependent HR we first assessed transgene copy number. Analysis of the six positive transformants indicated that transformant PS-2 has approximately 40 copies of the transgene, whereas all other transformants have 1–2 copies of the transgene (Table 1). To determine if the high copy number for PS-2 correlated with greater expression levels of *AvrSr50* early during the infection process, we inoculated Gabo wheat leaves with a single copy transformant (PS-7), double copy transformant (PS-22), the transformant containing 40 copies (PS-2) and the wild-type MoT N06047 strain. We extracted RNA at 3 dpi from three independent leaves for each MoT strain and conducted RNA-seq analysis. The levels of expression of *AvrSr50* were then analysed, illustrating that PS-2 had enhanced expression when compared to the single (PS-7) and double copy transformants (PS-22) (Fig. 2d). However, the increase in expression was not significant, although there was a general trend of increased expression with more *PWT3p::PWT3SP::AvrSr50* copies. In contrast, a control gene (MGG_01760—subunit of the exocyst complex) did not show increased expression in any of the transformants (Supplementary Figure. S6). We also assessed the expression levels of several genes encoding Avr and Avr homologues in the PS-2 transformant and wild-type MoT N06047 strain. This analysis illustrated that the expression of *AvrSr50* was similar in the PS-2 transformant to the expression of *AvrRmg8*, *AvrPizt*, *Avr1CO39* and *AvrPib* (Supplementary Figure. S7), indicating that the levels of *AvrSr50* expression in PS-2 were similar to those for endogenous MoT *Avr* genes.

**Table 1:** Transgene copy number of MoT transformants. Copy number analysis of transgenes was performed by iDNA genetics (Norwich, UK) using a TaqMan real-time PCR assay and a probe to detect the bialaphos resistance gene.

Construct	Transformant ID	HR on + *Sr50*	Copy No
pPWL2:AvrRmg8	PWLRmg-1	Y	1
	PWLRmg-3	N	1
	PWLRmg-5	Y	1
pPWL2:PWT3SP:AvrSR50	PWLS-2	N	1
	PWLS-8	N	21
	PWLS-11	N	1
pPWT3:PWT3SP:AvrSr50	PS-2	Y	40
	PS-7	N	1
	PS-10	N	2
	PS-22	N	2
	PS-35	N	3
	PS-41	N	1
	PS-42	N	1
	PS-43	N	1
	PS-44	N	1
pRP27:PWT3SP:AvrSr50	RS-2	N	1
	RS-6	N	4
pTrpc:PWT3SP:AvrSr50	TS-7	N	2
	TS-11	N	1

*HR* hypersensitive response.

To further explore the potential increase in expression of *AvrSr50* in the PS-2 transformant, we repeated the infection assays on the wheat line Gabo with the single copy (PS-7) and 40 copy (PS-2) transformants and performed RT-qPCR experiments at 1, 2, 3, 4 and 5 dpi. We found that the 40 copy transformant (PS-2) had consistently higher levels of *AvrSr50* expression across all time-points, with a statistically significant increase at 2 dpi when compared to the single copy transformant (PS-7) (Fig. 2e). This analysis further supports that the level of *AvrSr50* expression correlates with the number of *PWT3p::PWT3SP::AvrSr50* transgenes introduced into MoT and indicates that MoT may be useful as an effective surrogate delivery system for fungal avirulence effectors in wheat if sufficient expression of the transgene is obtained.

### The MoT PS-2 transformant contained a single tandem insertion of the transgene near a region of known structural variation

In addition to transgene copy number the location of inserted transgenes can affect transgene expression levels^27^. To assess the location of the transgene in the MoT PS-2 transformant, we conducted long read genome sequencing of the PS-2 transformant using nanopore technology. A total of 6.3 Gbp of total base counts were obtained, equating to approximately 153 × coverage for the predicted 41 Mb genome, with an N50 of 24,624 bp. This was comparable to data generated from conducting genome sequencing of other MoT isolates using similar methods^28^ (Supplementary Table S1).

**Figure 3:**
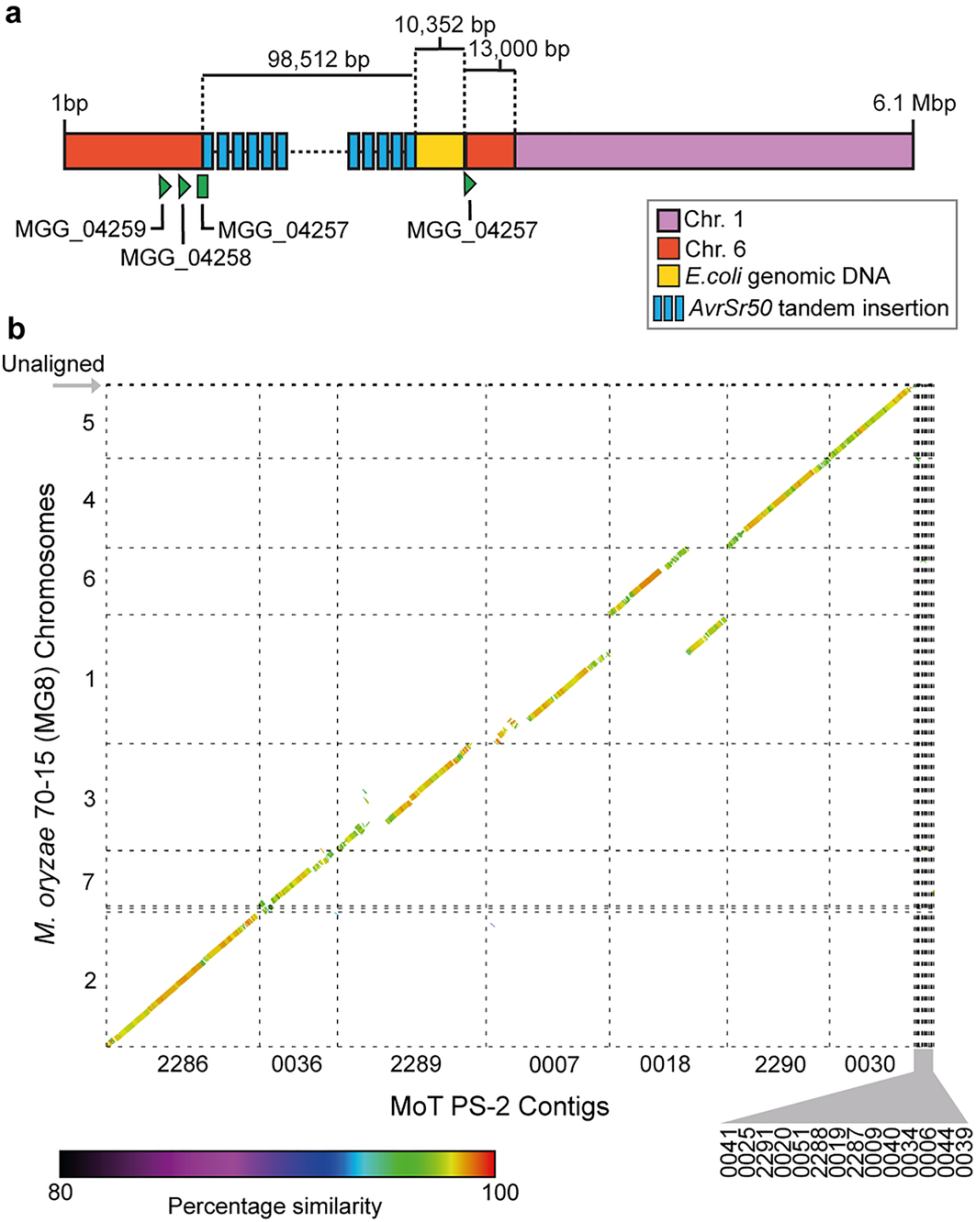
A single tandem insertion of the *AvrSr50* transgene occurred in the MoT PS-2 transformant, near a region of known structural variation. (**a**) Schematic representation of the *PWT3p::PWT3SP::AvrSr50* transgene integration site in the MoT PS-2 transformant. Following sequencing and assembly of the PS-2 genome, BLAST searches of the resulting contigs were used to identify regions of similarity with the *M. oryzae* rice blast reference genome (70–15), *AvrSr50* construct and *Escherichia coli* genome. Green arrows indicate *M. oryzae* genes in the integration site that encode proteins with signal peptides in their N-terminus. Chr, chromosome. (**b**) Structural variation was evident between contig 0018 from the PS-2 genome assembly and chromosomes 1 and 6 of the MG8 *M. oryzae* rice blast reference genome of strain 70–15 as previously reported for MoT isolate B71^29^ (Supplementary Figure. S8). Syntenic dot plot comparison between MoT PS-2 and *M. oryzae* 70–15 genomes shows genome order, with points coloured by sequence similarity. Alignments between the assemblies were performed using Nucmer and those with at least a 10 Kb match and 80% identity are shown.

Following assembly of the PS-2 genome into 21 contigs (Supplementary Table S2), we performed a BLAST search to identify the location of the multiple *PWT3p::PWT3SP::AvrSr50* transgenes using the expression vector as a query. We located a single tandem insertion of *PWT3p::PWT3SP::AvrSr50* within contig 0018, which included the entire donor vector concatenated multiple times with a total length of 98,512 bp (Fig. 3a). We also identified 10,352 bp of *E. coli* genomic DNA that had integrated into the insertion site, immediately downstream of the *PWT3p::PWT3SP::AvrSr50* tandem insertion. The insertion event occurred directly downstream of the homolog of the MGG_04257 gene, which is located on chromosome 6 of the rice blast reference genome (Fig. 3a). It is possible that the MGG_04257 promoter could be responsible for driving high levels of expression of the first copy of the tandem insertion. To assess this, RNA-seq reads generated at 3 dpi for PS-2 were de novo assembled and those containing the *AvrSr50* coding sequence searched for presence of the 5’UTR originating from MGG_04257. No reads were identified that contained both the *AvrSr50* sequence and 5’UTR originating from MGG_04257, indicating that the *PWT3* promoter was likely driving *AvrSr50* transcription in the PS-2 transformant.

The *PWT3p::PWT3SP::AvrSr50* insertion site in MoT PS-2 was also within close proximity to a known region of structural rearrangement that has been previously noted when comparing MoT isolate B71 with the rice blast pathogen MG8 assembly of *M. oryzae* strain 70–15^29,30^. We further confirmed this rearrangement in the PS-2 transformant of MoT strain N06047 through co-linearity analysis. Chromosomes from the *M. oryzae* MG8 genome assembly were aligned to the MoT PS-2 contigs (Fig. 3b), with the region containing the transgene insertion identified on contig 0018 that aligned to chromosome 6 in the MG8 assembly. In addition, a large portion downstream of the insertion site in PS-2 aligned to chromosome 1 of the *M. oryzae* MG8 assembly (Fig. 3a,b). Furthermore, alignment of the MoT PS-2 genome assembly to the MoT isolate B71 assembly showed complete co-linearity with B71 chromosome 6 (Supplementary Figure. S8). This suggests that the *PWT3p::PWT3SP::AvrSr50* transgene in PS-2 inserted into a region of known structural re-arrangement in *M. oryzae*.

### Targeted insertion of a single copy of *AvrSr50* into the MGG_04257 locus did not lead to a *Sr50-*dependent HR phenotype

**Figure 4:**
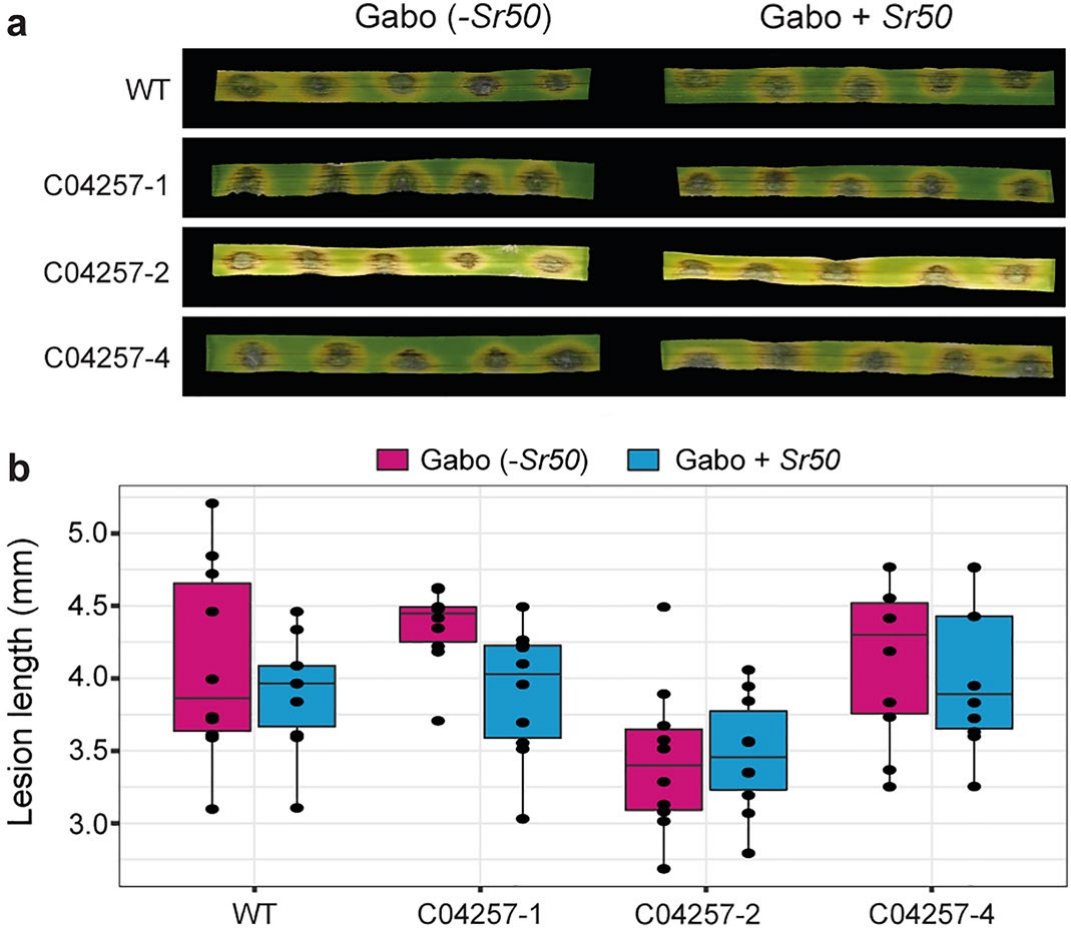
Integration of *AvrSr50* into the MoT MGG_04257 locus did not lead to a *Sr50*-dependant HR phenotype. (**a**–**b**) Introduction of *PWT3p::PWT3SP::AvrSr50* into the MoT MGG_04257 locus did not lead to a visible *Sr50*-mediated hypersensitive response (HR), for the three MoT transformants analysed (C04257-1, C04257-2, C04257-4). The second leaf of two-week old wheat seedings of the lines Gabo (*Sr50-*) and Gabo + *Sr50* were inoculated with conidial suspensions from each of the three separate transformants and wild-type (WT) MoT strain (N06047) using the spot inoculation method. Images were taken and lesion lengths measured at 4 days post-inoculation, with three biological replicates conducted (separate leaves). No statistical difference was evident (2-tailed *t*-test).

It is possible that genomic location and/or copy number is important for obtaining sufficient expression of *AvrSr50* in the MoT PS-2 transformant to elicit the observed visible *Sr50*-dependant HR phenotype. To determine if genome location alone was leading to the high levels of transgene expression in MoT PS-2, we targeted a single copy of the *PWT3p::PWT3SP::AvrSr50* transgene to the MGG_04257 locus using CRISPR/Cas9^31^. The donor DNA was cloned to have homology either side of the predicted cut site, which is usually 3–4 bp upstream of the protospacer adjacent motif (PAM) NGG sequence (Supplementary Figure. S9). The lengths of the homology arms (701 bp for the 5’ arm, and 404 bp for the 3’ arm) corresponded to the maximum length on either side of the cut site before encountering type II restriction sites used in golden gate cloning. A total of three transformants were confirmed by PCR and all transformants selected for further analysis. The three transformants (C04257-1, C04257-2 and C04257-4) were used for infection of leaves from two-week old seedlings of the two wheat lines differential for *Sr50*. At 5 dpi, there was no visible evidence of *Sr50-*dependent HR for the three tested *PWT3p::PWT3SP::AvrSr50* transformants (Fig. 4a,b and Supplementary Figure. S3). This data illustrates that introduction of a single copy of *PWT3p::PWT3SP::AvrSr50* at the MGG_04257 locus was insufficient to elicit an *Sr50*-dependent HR phenotype.

### Introduction of *AvrPm3* into MoT did not elicit a* Pm3*-mediated HR phenotype

**Figure 5:**
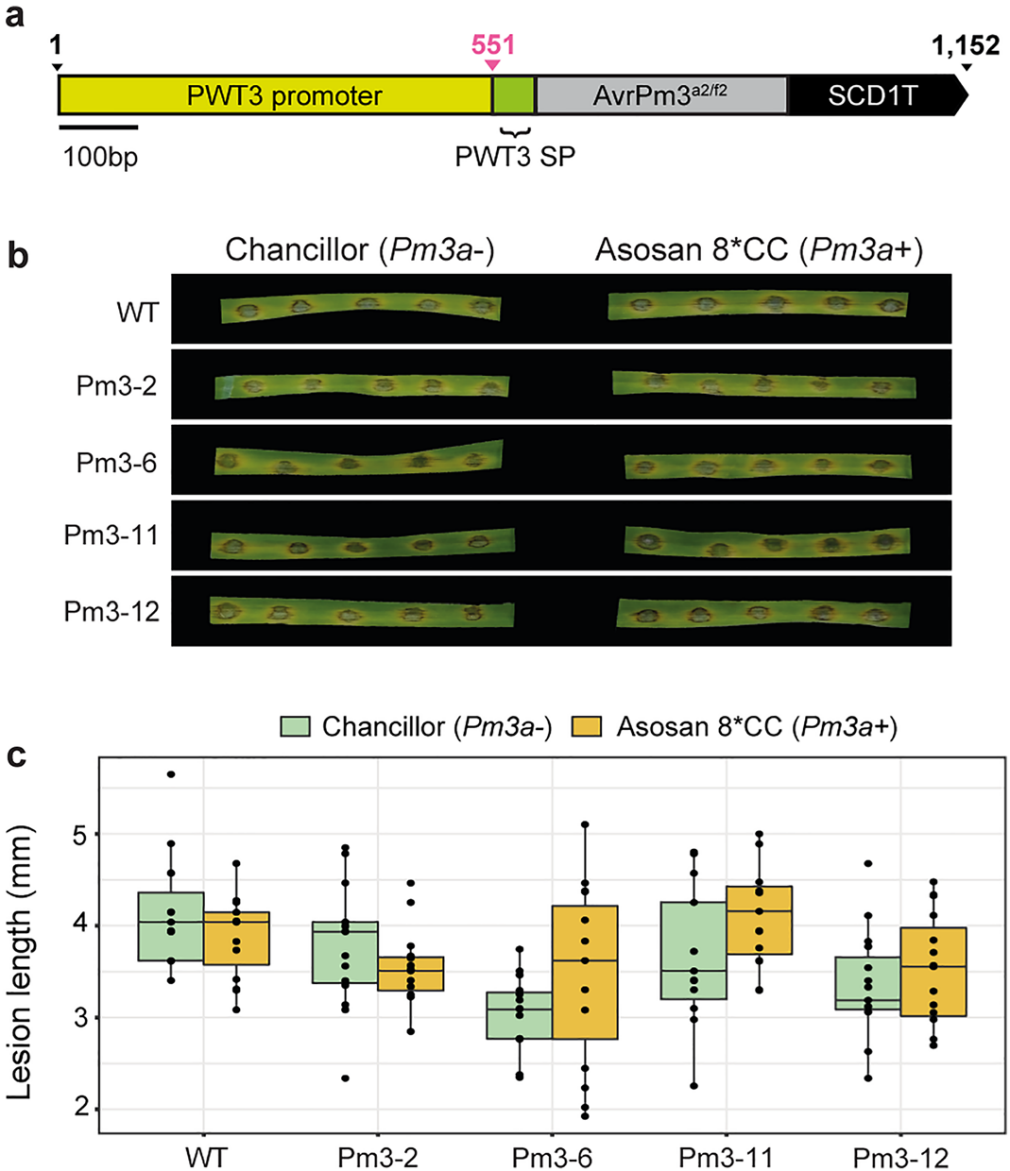
Introduction of the *Blumeria graminis* avirulence effector *AvrPm3* into MoT did not lead to a visible *Pm3*-dependant HR phenotype. (**a**) Schematic representation of the pCB-PWT3p-PWT3SP-AvrPm3^a2/f2^*-*stop vector used for transformation of MoT (strain N06047). Scale bar represents 100 bp; SP, signal peptide. (**b,c**), Introduction of the *PWT3p::PWT3SP:: AvrPm3*^*a2/f2*^ transgene into MoT failed to induce a visible *Pm3*-mediated hypersensitive response (HR), when transformants were inoculated onto the wheat line Asosan 8*CC (*Pm3* +). The second leaf of two-week old wheat seedings of the lines Asosan 8*CC (*Pm3* +) and Chancillor (*Pm3-*) were inoculated with conidial suspensions derived from four separate transformants (Pm3-2, Pm3-6, Pm3-11 and Pm3-12) and the wild-type (WT) MoT strain N06047 using the spot inoculation method. Images were taken and lesion lengths measured at 4 days post-inoculation, with three biological replicates conducted (separate leaves).

It is possible other Avr/R pairs may require less *Avr* expression for the elicitation of a visible HR phenotype^14,32^. To test this possibility, we selected the *AvrPm3*^*a2/f2*^ avirulence effector from *Blumeria graminis* f. sp *tritici* for analysis^33^. First, the 321 bp C-terminal effector domain of *AvrPm3*^*a2/f2*^ was cloned downstream of the *PWT3* promoter and *PWT3* SP (Fig. 5a). The resulting pCB-PWT3p-PWT3SP-AvrPm3^a2/f2^*-*stop vector was then transformed into MoT strain N06047. A total of four independent transformants were confirmed by PCR and used in infection assays alongside the wild-type MoT strain N06047. Two-week old wheat seedlings of lines Asosan 8*CC (*Pm3a* +) and Chancillor (*Pm3a-*)^34^ were inoculated and at 5 dpi no evidence of a visible *Pm3a*-dependant HR phenotype was evident for any of the four *PWT3p::PWT3SP:: AvrPm3*^*a2/f2*^ transformants (Fig. 5b and Supplementary FigURE. S10). Disease lesions observed on the *Pm3a* + line was comparable between the transformants and the wild-type MoT strain (N06047) and to plants lacking *Pm3a* (Fig. 5c). This indicates that the avirulence properties of *AvrPm3*^*a2/f2*^ could not be detected when *AvrPm3*^*a2/f2*^ was integrated into MoT under the *PWT3* promoter and *PWT3 SP*.

## Discussion

In this study, we uncovered the potential for MoT to act as an effective heterologous expression and delivery system for avirulence proteins in wheat, when using the *Pgt* effector AvrSr50 as a proof-of-concept. MoT is particularly attractive as a surrogate system for effector functional analysis in wheat due to the plethora of genetic resources readily available for the closely related rice blast pathogen; a long-standing model organism for studying plant-pathogen interactions^35^. The biotrophic growth phase of MoT also parallels the obligate biotrophic lifestyle of *Pgt*, with both fungi infecting wheat as their primary host and producing specialised infection structures for effector delivery and nutrient uptake from the plant^36^. However, the broader utility of MoT as a heterologous expression system could potentially be limited in certain countries due to its classification as a quarantine pathogen. MoT first emerged in South America in the 1980’s and remained constrained to this region until 2016 when it was identified for the first time in Asia in Bangladesh^37^, and in 2018 in Zambia^38^. Hence, due to its narrow geographic distribution and lack of effective control measures, MoT is classed as a quarantine pathogen in many locations worldwide and its utility as a surrogate expression system should be guided by country-specific regulatory measures.

We also found that MoT may only be useful as an effective surrogate delivery system when sufficient expression of the transgene is obtained; only a single *AvrSr50* transformant with very high copy number and transcript abundance, was found to elicit an *Sr50-*dependent HR. In addition, although several promoter and signal peptide combinations were assessed, only when using those from the MoT *PWT3* effector^25^ was an *Sr50-*dependent HR evident. The alternative constitutive promoters (*RP27* and *TrpC)* and *PWL2* promoter selected, have been used successfully for ectopic expression of transgenes in *Magnaporthe*^26,39^*.* Furthermore, the *RP27* promoter was used previously for delivery of *AVR1–CO39*^40^ and *PWL2* promoter for delivery of fluorescent protein fusion constructs that accumulated at the biotrophic interfacial complex (BIC) and were subsequently translocated into rice host cells^23^. This suggests that these promoters are suitable for targeting effectors to the rice host cytoplasm. However, their utility in the MoT-wheat pathosystem remains unclear given the absence of a *Sr50*-mediated HR response in our experiments. It is possible that the low copy number of the resulting transformants and expression levels could have led to an absence of *R* gene dependent HR. However, we did note one transformant obtained when using the *PWL2* promoter and *PWT3* signal peptide (termed PWLS-8) had 21 copies of *AvrSr50* and yet was still unable to elicit an *R* gene dependent HR, although a slight reduction in lesion size was noted in + *Sr50* plants (Supplementary Figure. S2).

In fungi, transgene copy number can positively or negatively affect transgene expression. The high levels of *AvrSr50* expression in transformant PS-2 correlated with integration of 40 copies of the transgene, all tandemly inserted into the genome. During protoplast transformation, it is common to obtain copy number variants^41^, however such high copy insertions, especially tandem insertions on this scale, are rare. The integration of multi-copies of a transgene are often the result of several copies entering a single nucleus and recombination prior to genomic integration into a single locus, frequently leading to higher levels of transgene expression^42^. However, in fungi and plants, integration of multiple transgene copies can also suppress transgene expression with varying degrees of silencing, a phenomenon that has been well studied and termed ‘quelling’ in *Neurospora* where it occurs at the post-transcriptional level^43^. In our case, we found no evidence of transgene silencing in multi-copy transformants, with visual symptoms of *Sr50*-mediated HR only clearly evident, when a high number of transgene copies were obtained.

Variation in transgene expression can also be strongly influenced by the insertion site. For transformant PS-2, the tandem multi-copy insertion of *AvrSr50* was found in a region equivalent to chromosome 6 in the MoT B71 genome. This region is close to a location of known structural variation in the *M. oryzae* 70–15 MG8 genome assembly^29^, with transposable elements playing a crucial role in driving plasticity of this region^44^. The tandem insertion of *AvrSr50* in transformant PS-2 was also found to disrupt the homolog of the rice blast gene termed MGG_04257, which is known to be upregulated during rice infection, encodes a canonical signal peptide at the N-terminus and is predicted to be a secretory lipase^45^. With two further neighboring genes (MGG_04258 and MGG_04259) displaying characteristics of effector proteins, this location is thus likely transcriptionally active during early time points of infection, which could have contributed to elevating *AvrSr50* expression in the PS-2 transformant. However, targeting a single copy of the *AvrSr50* transgene to the MGG_04257 locus using CRISPR/Cas9 failed to generate transformants that could elicit *Sr50* dependent HR. Therefore, it seems likely that the high number of transgene insertions had the greater influence on *AvrSr50* abundance and subsequent HR induction.

We found that only with very high *AvrSr50* copy number and transcript abundance, can an *Sr50-*dependent HR be initiated. To enhance expression of *AvrSr50* in a reproducible manner, further optimisation of the MoT system is required to consistently elevate the number of transgene copies integrated or enhance expression of the transgene by targeting transgenes to locations of high transcriptional activity and/or through synthetic design. For instance, variation of the terminator sequence, development of synthetic promoters and transcriptional regulators have all been utilised to control and enhance production of gene expression cassettes^42^. If these limitations can be overcome, this would present MoT as an ideal accessible fungal surrogate system for studying the virulence/avirulence function of candidate effectors in future, particularly from intractable obligate biotrophic pathogens that are recalcitrance to genetic transformation.

## Materials and methods

### Fungal growth and transformation

MoT strains were routinely maintained on complete media^46^ with 1.5% agar or cultured in liquid complete media with agitation (150 rpm) at 24 °C for 48 h. Protoplast-mediated transformation of MoT (strain N06047) was performed as previously described^46^, with resulting transformants selected on glufosinate (40 μg/mL). Genomic DNA was extracted from MoT transformants for genotypic analysis and nanopore sequencing using the CTAB method^46^. Copy number analysis of transgenes was performed by iDNA genetics (Norwich, UK) using a TaqMan real-time PCR assay and a probe to detect the bialaphos (BAR) resistance gene. A full list of MoT strains generated is provided in Supplementary Table S3.

### MoT infection assays

Seeds from bread wheat (*Triticum aestivum*) lines Vuka, S-615, Gabo, Gabo + *Sr50*, Chancillor and Asosan 8*CC were pre-germinated, sown in cell trays and grown in a controlled environment consisting of long-day conditions (16-h light/8-h dark) under a 19 °C/14 °C temperature cycle (day/night). When seedlings reached two-week stage, plants were subjected to MoT inoculation using either detached leaf spot inoculations or spray inoculations^47^. In short, conidial suspensions of 1 × 10^5^ spores/mL of each MoT isolate were prepared in 0.25% (v/v) tween gelatine, with 5 μL droplets added for spot inoculations with wicking of droplets after 24 h^47^. Immediately following inoculation, plants were incubated in high humidity and dark conditions for 24 h before being returned to controlled environment conditions. Infection phenotypes were assessed 4–5 dpi, with lesion lengths measured in Image J and assessed using a student’s *t*-test. All experiments were performed in accordance with relevant guidelines and regulations.

### Expression vectors for MoT transformation

The avirulent 330 bp allele of *AvrRmg8* was PCR amplified from genomic DNA of MoT (strain BTJP4-1) using primers AvrRmg8_BamH1F and AvrRmg8_EcoRV_R and introduced into the pCB-Ppwl2-mcherry-stop vector^21^. The 333 bp *AvrSr50* sequence (devoid of signal peptide region) was synthesized by Genscript (USA) and PCR amplified from the resulting pUC vector and the 54 bp MoT *PWT3* signal peptide region was PCR amplified from genomic DNA of MoT (strain N06047). The *PWT3* signal peptide and *AvrSr50* coding sequence (devoid of the signal peptide) were subsequently joined by ‘splicing by overlap extension’ PCR^48^. Promoter sequences from *RP27* (478 bp) and *PWT3* (550 bp) from MoT and pTrpC (357 bp) from *Aspergillus nidulans* were PCR amplified from MoT genomic DNA (strain N06047) and vector pBHt2G-RFP (Addgene, USA) respectively, using primers pRP27-Not1F and pRP27-Xba1R, pPWT3-Not1F and pPWT3-Xba1R, and pTrpc-Not1F and pTrpc-Xba1 (Supplementary Table S4). The resulting amplicons were independently introduced into pCB-Ppwl2-mcherry-stop vector^21^ followed by introduction of *PWT3SP*::*AvrSr50*.

The pCB-PWT3p-PWT3SP-AvrPm3^a2/f2^*-*stop vector was generated using golden gate cloning. Level zero components were generated for the 550 bp *PWT3* promoter, *PWT3* signal peptide region (54 bp), and 321 bp *AvrPm3*^*a2/f2*^ coding sequence (without the native signal peptide). The *AvrPm3*^*a2/f2*^ coding sequence was PCR amplified from genomic DNA of the avirulent *Blumeria graminis* f. sp *tritici* strain 96224^33^ using primers AvrPm3a_GGF and AvrPm3a_GGR and the transcription terminator of *SCD1* from pCB-Ppwl2-mcherry-stop using primers 3SCD1T_GGF and 3SCD1T_GGR. Level 0 constructs were then integrated into the pcb-1532B level 1 vector^49^. Primer sequences are provided in Supplementary Table S4.

### CRISPR/Cas9 targeted insertion of AvrSr50 into MoT

To insert the *PWT3p::PWT3SP::AvrSr50* transgene into the MGG_04257 locus, a suitable protospacer adjacent motif (PAM) sequence within the homolog of MGG_04257 in MoT was selected. The MGG_04257 locus was analyzed in the genome sequence of MoT strain Br32 and that generated herein for MoT transformant PS-2, with a PAM sequence at position 803 selected due to the position giving the highest activity and specificity score^50^ in MGG_04257. Homologous sequence either side of the PAM sequence (701 bp for the 5’ arm, and 404 bp for the 3’ arm) were amplified from MoT genomic DNA (strain N06047) using PCR and primers 5′04257_GGF and 3′04257_GGF, and 5′04257_GGR and 3′04257_GGR (Supplementary Table S4). The resulting amplicons were cloned into the universal acceptor plasmid pUAP1^51^. The *AvrSr50* coding sequence was domesticated to remove *BsaI* and *BpiI* sites using the Q5 site directed mutagenesis kit (New England Biolabs, USA) and primers AvrSr50_Q5_F and AvrSr50_Q5_R. A level zero construct containing the BAR resistance gene was constructed by PCR amplifying Pcb-pPWL2-mcherry vector^21^ with primers BAR_GGF and BAR_GGR and cloning the amplicon into pUAP1^51^. Finally, level zero constructs containing the 5’ and 3’ homologous regions of MGG_04257, the *PWT3* promoter, fusion of *PWT3SP:AvrSr50*, *SCD1* transcription terminator, and BAR resistance gene were assembled into the level 1 acceptor vector pICH47732^51^.

The single guide RNA (sgRNA) was designed using Geneious prime 2022.1.1 (Biomatters Ltd, New Zealand; Supplementary Figure S9), sgRNAs were synthesized using the EnGen sgRNA synthesis kit (New England Biolabs, USA) following the manufacturer’s instructions. Immediately after synthesis, sgRNAs were purified using a T2040 RNA clean-up kit (New England Biolabs, USA) and sgRNAs were then complexed with 6 μg purified Cas9 (New England Biolabs, USA) at a 1:1 molar ratio for 10 min at room temperature. During protoplast transformation, appropriate donor DNA (2 μg in 6 μL) was added alongside pre-complexed Cas9/sgRNA (4 μL). Transformants were confirmed by PCR using primers MGG_04257_F2 and AvrSr50_RTR1 (Supplementary Table S4) that annealed upstream the 5’ homology region or the AvrSr50 gene, respectively.

### RNA-seq analysis

Total RNA was extracted from MoT infected leaf material collected 3 dpi using the RNeasy Plant Mini Kit (Qiagen, Germany). RNA was sent to GENEWIZ (UK) for cDNA library preparation and sequencing conducted on the Illumina HiSeq 2500 platform. The resulting 150 bp, paired end reads were quality trimmed and filtered using Trimmomatic version 0.39 ^52^ and transcript abundances (TPM values; transcript per million) quantified following pseudoalignment to the MoT reference transcriptome, strain B71^29^, with the *AvrSr50* transcript added using Kallisto v 0.46^53^. De novo assembly of transcripts was performed using Trinity v2.11.0^54^ and used to search for the *AvrSr50* coding sequence with BLASTN^55^.

### RT-qPCR analysis of *AvrSr50* gene expression

The second leaf from three-week-old Gabo plants were spray inoculated with transformants PS-7 and PS-2 as described above. Leaves were collected at 1–5 dpi and flash frozen in liquid nitrogen followed by total RNA extraction using the RNeasy Plant Mini Kit (Qiagen, Germany). Genomic DNA contamination was removed using a TURBO DNA-free Kit (Ambion, UK) and RNA concentration determined using a Qubit Fluorometer (ThermoFisher, USA). First-strand cDNA was synthesised using SuperScript™ II Reverse Transcriptase (Invitrogen, USA), using 3 μg of RNA, random hexamers and Oligo(dT) primers according to the manufacturer’s instructions. RT-qPCR was performed on a LightCycler 480 (Roche, Switzerland) using LightCycler 480 SYBR Green I Master Mix (Roche, Switzerland) following the manufacturer’s instructions with each primer at a final concentration of 0.25 μM (Supplementary Table S5). Three technical replicates were prepared per reaction and *AvrSr50* expression was compared to actin (MGG_03982) as a reference.

### Assembly of the MoT PS-2 genome and collinearity analysis

DNA was extracted from fungal material of MoT transformant PS-2 grown on CM agar plates using the CTAB method^46^ with the length of DNA fragments assessed using the Femto Pulse System (Agilent, USA), DNA purity using a Nanodrop (ThermoFisher, USA) and concentration using the Qubit Fluorometer (ThermoFisher, USA). Libraries were constructed using 4 μg of genomic DNA and the 1D genomic DNA by ligation kit (SQK-LSK109; Oxford Nanopore Technologies, UK). The DNA sample was sequenced using the MinION sequencer with FLO-MIN106D R9 flow cells (Oxford Nanopore Technologies, UK) following the manufacturer’s instructions. Base calling and demultiplexing was performed using Albacore v.2.3.3 (Oxford Nanopore Technologies, UK) and reads de novo assembled into contigs using Canu v1.8^56^. Contigs derived from the PS-2 assembly were aligned to the MoT genome assembly of strain B71^29^ and the rice blast pathogen MG8 genome assembly of *M. oryaze* strain 70–15^30^ using the NUCmer utility of the MUMmer3 software^57^. The coordinate output file was filtered for sequence alignments > 10 kb in length, with > 70% similarity (delta-filter options -l 10,000 -i 70). The MUMmerplot utility was used to generate a dot plot for visualization (options -l and–color). The colour scale was adjusted to display similarity between 80 and 100%.

## Supplementary Information

Supplementary Information.

## Data Availability

Sequence data that support the findings of this study can be found in the European Nucleotide Archive (ENA) database under the following accession number: PRJEB55233 (https://www.ebi.ac.uk/ena/browser/view/PRJEB55233).
