# Supplementary material for: Magnaporthe oryzae pathotype Triticum (MoT) can act as a heterologous expression system for fungal effectors with high transcript abundance in wheat
Source: Sci Rep. 2023 Jan 3;13:108. doi: 10.1038/s41598-022-27030-z (PMC9810704; doi:10.1038/s41598-022-27030-z)
Supplement: Supplementary file 1 — Supplementary Information. [file 41598_2022_27030_MOESM1_ESM.pdf]

Supplementary Information

Magnaporthe oryzae pathotype *Triticum* (MoT) can act as a heterologous expression system for fungal effectors with high transcript abundance in wheat

Cassandra Jensen, Diane G.O. Saunders

The following Supplementary Information is available for this article:

Fig. S1. MoT strain N06047 carries a previously uncharacterised *AvrRmg8* allele.

Fig. S2. Introduction of *Pgt AvrSr50* into MoT under the *PWL2* promoter failed to induce a visible *Sr50*-mediated HR phenotype.

Fig. S3. Introduction of *PWT3p::PWT3SP::AvrSr50* into MoT induced a visible *Sr50*-mediated hypersensitive response (HR) for one transformant (PS-2).

Fig. S4. *Sr50*-mediated HR was not evident after inoculation with MoT transformants harbouring *RP27p::PWT3SP::AvrSr50* or *TrpCp::PWT3SP::AvrSr50.*

Fig. S5. A single transformant (PS-2) expressing *AvrSr50* under the *PWT3* promoter and signal peptide can elicit *Sr50*-dependent HR.

Fig. S6. Expression of the gene MGG_01760, that encodes a subunit of the exocyst complex, was comparable for all *PWT3p::PWT3SP::AvrSr50* MoT transformants assessed.

Fig. S7. Levels of expression of the MoT effector genes *AvrRmg8*, *AvrPizt*, *Avr1CO39* and *AvrPib* was comparable to *AvrSr50* expression in the MoT transformant PS-2.

Fig. S8. The PS-2 genome assembly is colinear with the genome assembly of MoT strain B71.

Fig. S9. Schematic of the *PWT3p::PWT3SP::AvrSr50* transgene cassette targeted to the MGG_04257 locus in MoT using CRISPR/Cas9.

Fig. S10. Introduction of the *Blumeria graminis* avirulence effector *AvrPm3* into MoT did not lead to a visible *Pm3*-dependant HR phenotype.

Table S1. Summary statistics of nanopore reads following sequencing of various MoT strains.

Table S2. Summary statistics of MoT genome assemblies generated following nanopore sequencing.

Table S3. List of MoT strains generated in this study.

Table S4. Primers used for cloning and genotyping MoT strains.

Table S5. Primers used for RT-qPCR assays.

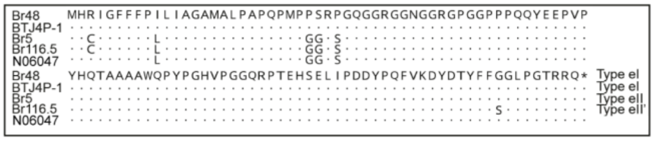

Fig. S1. MoT strain N06047 carries a previously uncharacterised *AvrRmg8* allele. Multiple sequence alignment of the protein sequences of the known *AvrRmg8* alleles and the allele found in MoT strain N06047. Br48, BTJ4P-1, Br5 and Br116.5 are MoT strains; Type eI, Type eII, Type eII' are previously described alleles of *AvrRmg8*[1].

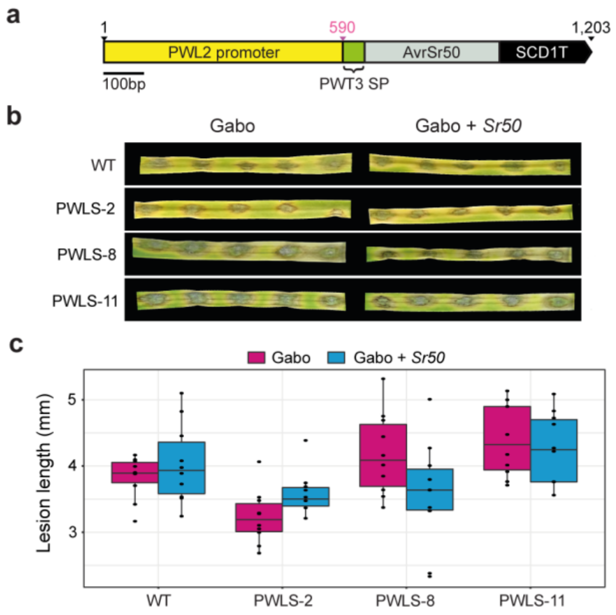

Fig. S2. Introduction of *Pgt AvrSr50* into MoT under the *PWL2* promoter failed to induce a visible *Sr50*-mediated HR phenotype. a, Schematic representation of the pCB-Ppwl2-PWT3SP-AvrSr50-stop vector used for transformation of MoT (strain N06047). Scale bar represents 100 bp; SP, signal peptide. **b-c,** Introduction of *PWL2p::PWT3SP::AvrSr50* into MoT failed to induce a visible *Sr50*-mediated hypersensitive response (HR) when transformants were inoculated onto the wheat line Gabo + *Sr50*. Conidial suspensions from the MoT wild type (WT) strain N06047 and three independent MoT transformants (PWLS-2, PWLS-8 and PWLS-11) were inoculated onto the second leaf of two-week old wheat plants (lines Gabo [*Sr50*-] and Gabo + *Sr50*). Inoculations were performed using the spot inoculation method and three biological replicates (separate leaves) were assessed. Images were taken and lesion lengths analysed at 4 days post-inoculation.

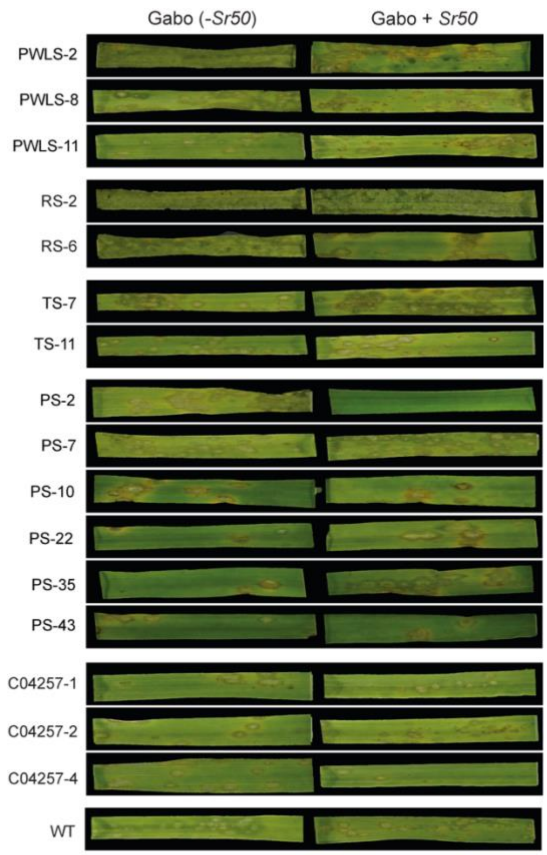

Fig. S3. Introduction of *PWT3p::PWT3SP::AvrSr50* into MoT induced a visible *Sr50*-mediated hypersensitive response (HR) for one transformant (PS-2). Conidial suspensions from the MoT wild type (WT) strain N06047 and transformants expressing *AvrSr50* under various promoter and signal peptide combinations (PWLS-2, PWLS-8, PWLS-11, RS-2, RS-6, TS-7, TS-11, PS-2, PS-7, PS-10, PS-22, PS-35, PS-43, C04257-1, C04257-2, and C04257-4) were spray inoculated onto the second leaf of two-week old wheat plants (lines Gabo [*Sr50*-] and Gabo + *Sr50*). Three biological replicates (separate leaves) were assessed. Images were taken 5 days post-inoculation.

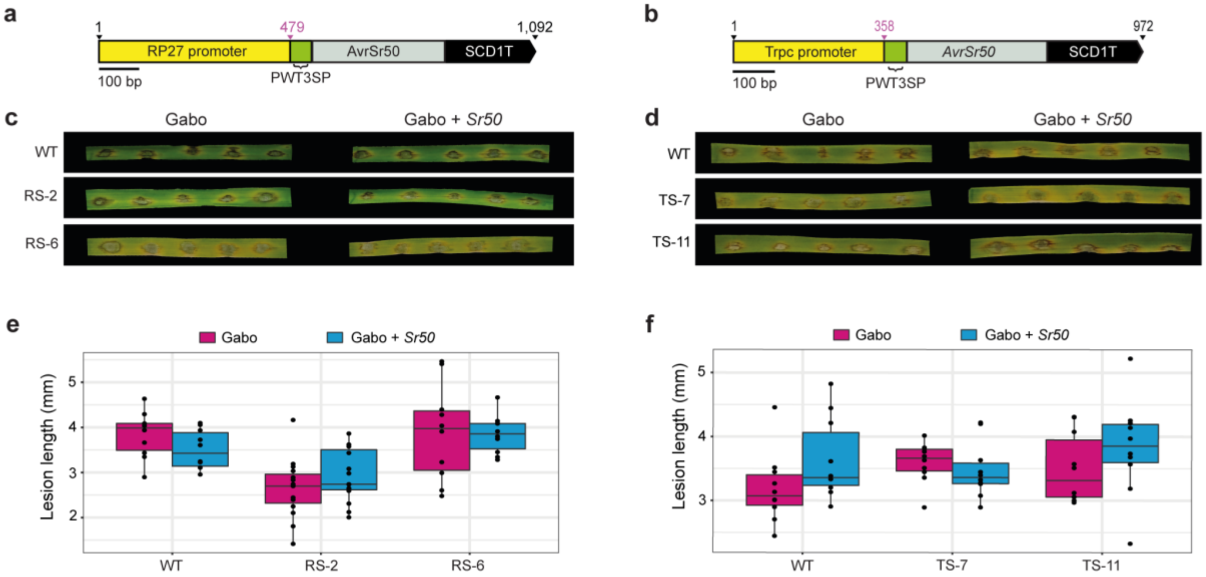

Fig. S4. *Sr50*-mediated HR was not evident after inoculation with MoT transformants harbouring *RP27p::PWT3SP::AvrSr50* or *TrpCp::PWT3SP::AvrSr50*. **a-b**, Schematic representations of the pCB-RP27p-PWT3SP-AvrSr50-stop and pCB-TrpCp-PWT3SP-AvrSr50-stop vectors used for transformation of MoT (strain N06047). Scale bar represents 100 bp; SP, signal peptide. **c-e,** Introduction of the *RP27p::PWT3SP::AvrSr50* and *TrpCp::PWT3SP::AvrSr50* transgenes into MoT failed to induce a visible *Sr50*-mediated hypersensitive response (HR) when transformants were inoculated onto the wheat line Gabo + *Sr50*. Conidial suspensions from the MoT wild type (WT) strain N06047 and each transformant were used to inoculate the second leaf of two-week old wheat seedings of the lines Gabo (-*Sr50*) and Gabo +*Sr50* using the spot inoculation method. Images were taken and lesion lengths measured at 4 days post-inoculation, with three biological replicates conducted (separate leaves).

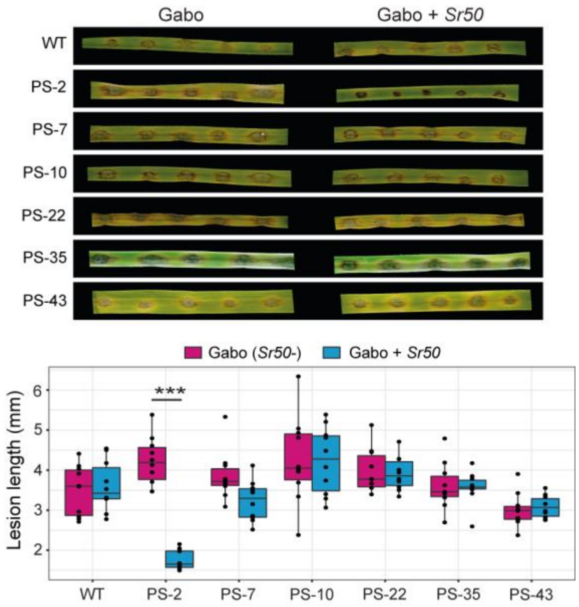

Fig. S5. A single transformant (PS-2) expressing *AvrSr50* under the *PWT3* promoter and signal peptide can elicit *Sr50*-dependent HR. Introduction of *PWT3p::PWT3SP::AvrSr50* into MoT induced a visible *Sr50*-mediated hypersensitive response (HR) for one transformant (termed PS-2), specifically restricting its growth when inoculated onto the wheat line Gabo + *Sr50*. Conidial suspensions from the MoT wild type (WT) strain N06047 and six *PWT3p::PWT3SP::AvrSr50* transformants were inoculated onto the second leaf of two-week old wheat plants (lines Gabo [*Sr50*-] and Gabo + *Sr50*) using the spot inoculation method and three biological replicates (separate leaves) assessed. Images were taken and lesion lengths analysed at 4 days post-inoculation. Asterisks denote statistically significant differences (***: $p < 0.001$; 2-tailed *t*-test).

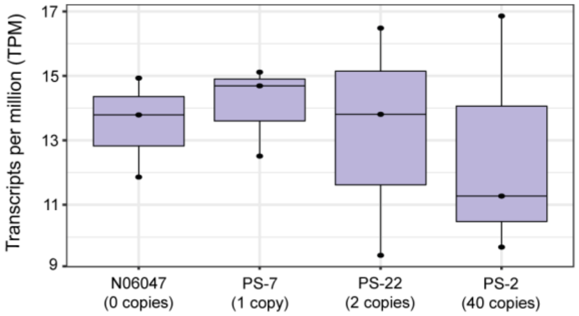

Fig. S6. Expression of the gene MGG_01760, that encodes a subunit of the exocyst complex, was comparable for all *PWT3p::PWT3SP::AvrSr50* MoT transformants assessed. MGG_01760 expression level was determined from RNA-seq analysis of wheat leaves (Gabo – *Sr50*) harvested 3 days post-inoculation with three independent transformants PS-7, PS-22, PS-2, and the MoT wild type (WT) strain N06047. Three biological replicates (separate plants) were analysed. Expression data is represented as Log_{10} TPM (transcripts per kilobase million). Isolates were infected on whole Gabo (*Sr50*-) plants and the second leaf was collected 3 days post-inoculation. Each data point represents a single biological replicate (separate plants and experiments). No statistical difference was evident (2-tailed *t*-test).

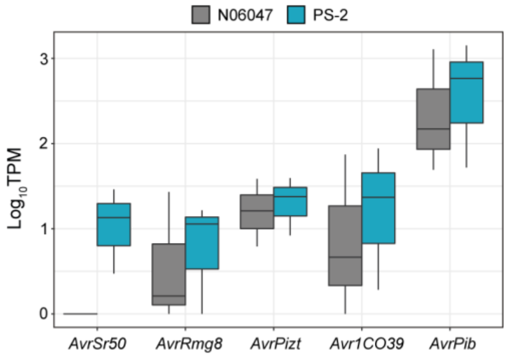

Fig. S7. Levels of expression of the MoT effector genes *AvrRmg8*, *AvrPizt*, *Avr1CO39* and *AvrPib* was comparable to *AvrSr50* expression in the MoT transformant PS-2. The expression levels of five genes encoding Avr and Avr homologues in MoT were determined from RNA-seq analysis of wheat leaves (Gabo –*Sr50*) harvested 3 days post-inoculation with the PS-2 transformant and WT strain N06047. Three biological replicates (separate plants) were analysed. Expression data is represented as Log_{10} TPM (transcripts per kilobase million). Isolates were infected on whole Gabo (*Sr50*-) plants and the second leaf was collected 3 days post-inoculation.

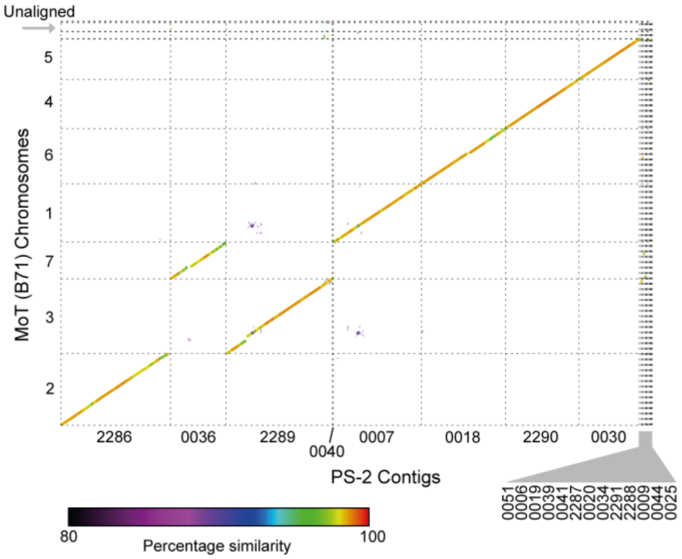

Fig. S8. The PS-2 genome assembly is colinear with the genome assembly of MoT strain B71. Syntenic dot plot comparison between the MoT PS-2 and B71 genome assemblies shows genome order, with points coloured by sequence similarity. Alignments between the assemblies were performed using Nucmer and those with at least a 10 Kb match and 80 % identity are shown.

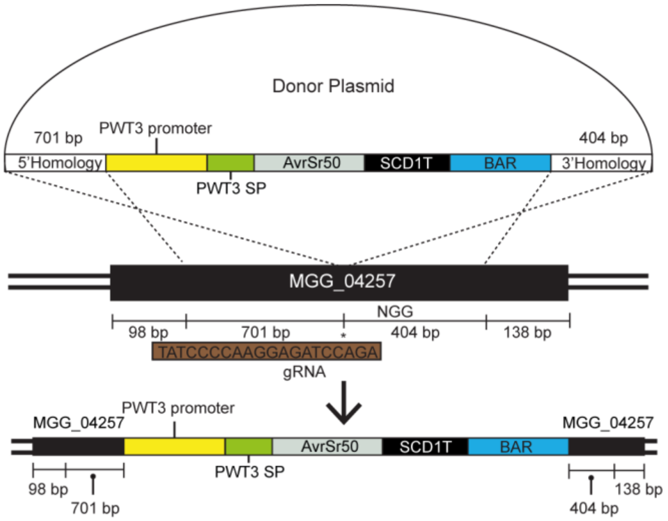

Figure S9. Schematic of the *PWT3p::PWT3SP::AvrSr50* transgene cassette targeted to the MGG_04257 locus in MoT using CRISPR/Cas9. The donor plasmid was cloned to contain the *PWT3p::PWT3SP::AvrSr50* transgene and 5'and 3' regions homology regions either side of the MGG_04257 locus insertion site. The gRNA (brown) was designed to target the region upstream of a protospacer adjacent motif (PAM) NGG sequence at position 803 in the MGG_04257 gene. The asterisk denotes the predicted cut site (~3bp upstream the PAM).

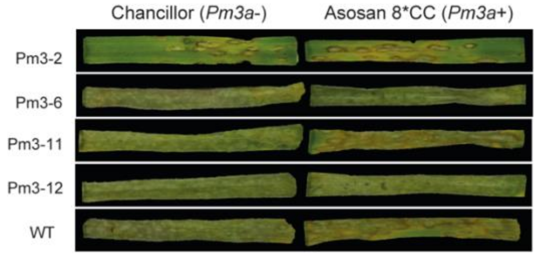

Fig. S10. Introduction of the *Blumeria graminis* avirulence effector *AvrPm3* into MoT did not lead to a visible *Pm3*-dependant HR phenotype. Conidial suspensions of four separate transformants (Pm3-2, Pm3-6, Pm3-11 and Pm3-12) and the wild-type (WT) MoT strain N06047 were spray inoculated onto the second leaf of two-week old wheat seedings of the lines Asosan 8*CC (*Pm3+*) and Chancillor (*Pm3-*). Images were taken at 5 days post-inoculation, with three biological replicates conducted (separate leaves).

Table S1. Summary statistics of nanopore reads following sequencing of various MoT strains.

MoT strain	No. of Reads	Base Count (Gbp)	Read N50 (bp)	Reference
PS-2	577, 392	6.3	24,624	This study
BTJP4-1	464,107	3.1	9,437	Win et al., 2019
BTMP13-1	181,812	1.4	25,365	Win et al., 2019
BTGP1-b	158,055	1.3	11,213	Win et al., 2019
BTGP6-f	284,212	1.9	9,448	Win et al., 2019
BR32	655,377	5.5	11,021	Win et al., 2019

Table S2. Summary statistics of MoT genome assemblies generated following nanopore sequencing.

MoT strain	Number of Contigs	Assembly Length (bp)	N50 (bp)	Max length (bp)	Mean Length (bp)	Min length (bp)
PS-2	21	42,866,477	6,394,504	7,937,122	2,041,260	44,244
BTJP4-1	59	44,506,712	4,344,896	7,174,201	754,351	13,054
BTMP13-1	16	43,978,087	6,037,509	10,783,101	2,748,630	7,390
BTGP1-b	74	44,406,102	2,814,025	6,505,875	600,082	5,533
BTGP6-f	57	44,234,333	3,705,381	6,048,575	776,041	8,312
BR32	21	41,471,325	5,047,693	11,366,628	1,974,825	18,099

Table S3. List of MoT strains generated in this study. All transgenes were introduced into MoT strain N06047.

**Transgenes**	**Transformant ID**	**Transgene description**
PWL2p::AvrRmg8	PWL8-1	*AvrRmg8* (with native signal peptide) and under the *M. oryzae PWL2* promoter.
	PWL8-3	
	PWL8-5	
PWL2p::PWT3SP::AvrSr50	PWLS-2	*AvrSr50* (without native signal peptide) and under the *M. oryzae PWL2* promoter and MoT *PWT3* gene signal peptide.
	PWLS-8	
	PWLS-11	
PWT3p::PWT3SP::AvrSr50	PS-2	*AvrSr50* (without native signal peptide) and under the MoT *PWT3* gene promoter and signal peptide.
	PS-7	
	PS-10	
	PS-22	
	PS-35	
	PS-41	
	PS-42	
	PS-43	
	PS-44	
RP27p::PWT3SP::AvrSr50	RS-2	*AvrSr50* (without native signal peptide), under the *M. oryzae* RP27 gene promoter and MoT *PWT3* gene signal peptide.
	RS-6	
TrpCp::PWT3SP::AvrSr50	TS-7	*AvrSr50* (without native signal peptide) under the *Aspergillus nidulans TrpC* gene promoter and MoT *PWT3* gene signal peptide.
	TS-11	
PWT3p::PWT3SP:: AvrPm3$^{a2/f2}$	Pm3-2	*AvrPm3* $^{a2/f2}$ (without native signal peptide) under the MoT *PWT3* gene promoter and signal peptide.
	Pm3-6	
	Pm3-11	
	Pm3-12	
PWT3p::PWT3SP::AvrSr50	C04257-1	*AvrSr50* (without native signal peptide) under the MoT *PWT3* gene promoter and signal peptide, targeted to the MGG_04257 gene locus.
	C04257-2	
	C04257-4	

Table S4. Primers used for cloning and genotyping MoT strains.

Primer Name	Sequence
AvrRmg8_P7	GCCATTTGCAGGATTGGTAT
AvrRmg8_P8	GATCGTTTCGAGCGTGATCT
AvrRmg8_BamH1F	AAGGATCCATGCACCGCATCGGCTTTTTCTTCC
AvrRmg8_EcoRV_R	AAGATATCCTACTGCCTTCTAGTACCGGGAAGT
pRP27-Not1F	AAGCGGCCGCATAAATGTAGGTATTACCTGTAC
pRP27-Xba1R	AATCTAGATTTGAAGATTGGGTTCCTAC
pPWT3-Not1F	AAGCGGCCGCGCTTTGCCGACTTTGGTAATAG
pPWT3-Xba1R	AATCTAGAAATGTTATATGTGCAAATATATATG
pTrpc-Not1F	AAGCGGCCGCAACTGATATTGAAGGAGCAT
pTrpc-Xba1R	AATCTAGATTGGATGCTTGGGTAGAATA
PWT3SP-BAMHI-F	AAGGATCCATGAACCTCAGACTTATTACTTTTTTA
PWT3SP-OverlapAvr-R	GACAAGGCTCCTAGCAGCCACCGCGCCG
AvrSr50-OverlapPWT3SP-F	GCCGGCGCGGTGGCTGCTAGGAGCCTTGTCAAAAT
AvrSr50-HindIII-R	AAAAGCTTCTACCTGTGTTGGCGCCTTG
pPWT3_GGF	AAGAAGACAACTCAGGAGGCTTTGCCGACTTTGGTA
pPWT3_GGR	AAGAAGACAACTCGCATTAATGTTATATGTGCAAATATATATGAG
PWT3SP_GGF	AAGAAGACAACTCAAATGAACCTCAGACTTATTACTTTTTTAATG
PWT3SP_GGR	AAGAAGACAACTCGTGCCACCGCGCCGGCCA
AvrPm3a_GGF	AAGAAGACAACTCAGGCAGGCCCTGTCGCTAACGCT
AvrPm3a_GGR	AAGAAGACAACTCGAAGCCTAGTGCAGAATTATGTTTAATTGAGG
3SCD1T_GGF	AAGAAGACAACTCAGCTTAGCGGCGTGCTCTGCACA
3SCD1T_GGR	AAGAAGACAACTCGAGCGCCGGGAGGCTGAATCGGA
AvrSr50_GGF	AAGAAGACAACTCAGGCAGCTAGGAGCCTTGTCAAA
AvrSr50_GGR	AAGAAGACAACTCGCGAACCCCTGTGTTGGCGCCTTGC
AvrSR50_Q5_F	ATTCACAAGTGTTCAATCATTTTG
AvrSr50_Q5_R	CAGCTTCAAACTCAGTGAG
5’04257_GGF	AAGAAGACAACTCAGGAGCGCCATCGCCACCGAGAACT
5’04257_GGR	AAGAAGACAACTCGGTCAGGATCTCCTTGGGGATAAGC
3’04257_GGF	AAGAAGACAACTCATCCCAGAGGGCCGGCCCGAACG
3’04257_GGR	AAGAAGACAACTCGAGCGGGCCTGGGGCACAGACAG
pPWT3_TGAC_GGF	AAGAAGACAACTCATGACGCTTTGCCGACTTTGGTA
SCD1T_TACT_GGR	AAGAAGACAACTCGAGTACCGGGAGGCTGAATCGGA

BAR_GGF	AAGAAGACAACTCATACTTCGACAGAAGATGATATT
BAR_GGR	AAGAAGACAACTCGGGGACAATGGGCTCGACCTAAA
MGG_04257_F2	GCGCTTTTGTATAAGAAGCTCAAGA
AvrSr50_RTR1	CGATTTCCTCATGTGGATTCC

Table S5. Primers used for RT-qPCR assays.

**Gene name**	**Genbank accession**	**Forward primer**	**Reverse primer**	**Efficiency**
M. oryzae Actin	XP_ 003719871	ACAATGGTTCGGGTATGTGC	CGACAATGGACGGGAAGAC	**85%**
Pgt AvrSr50	NA	ATGATGGACGTTCACCCTACATAG	CCTCATGTGGATTCCAAACAATCG	**101%**

References

Anh, V. L. *et al.* Rmg8 and Rmg7, wheat genes for resistance to the wheat blast fungus, recognize the same avirulence gene AVR-Rmg8. *Mol Plant Pathol* **19**, 1252-1256 (2018). https://doi.org:10.1111/mpp.12609

Win, J. *et al.* Nanopore sequencing of genomic DNA from *Magnaporthe oryzae* isolates from different hosts. *Zenodo* (2019). https://doi.org:10.5281/zenodo.2564950
